# 25. An interesting cause of foot drop

**DOI:** 10.1093/rap/rky033.016

**Published:** 2018-09-20

**Authors:** Gagandeep K Takhar, Andrew Porter, Simon Allard

**Affiliations:** Rheumatology Department, Chelsea & Westminster NHS Foundation Trust, Middlesex, UNITED KINGDOM

**Introduction:** Foot drop is an uncommon presentation on the acute medical take and to specialist clinics. This case describes an unusual cause of foot drop and highlights the need to consider a wide differential and start prompt treatment to prevent long term complications.

**Case description:** A 47 year old gentleman of Indian origin with a background of asthma diagnosed in childhood initially presented to the accident and emergency department with increasing shortness and a productive cough. There was no history of foreign travel or known TB contacts. He was treated for an infective exacerbation of asthma with nebulisers, steroids, antibiotics and discharged. He was re-admitted to hospital a few weeks later with general malaise, a pruritic rash, shortness of breath, leg pain and evidence of left sided foot drop. CT Chest showed pleural effusions in conjunction with a pericardial effusion. Blood tests showed raised inflammatory markers, an eosinophilia with a negative ANA, ANCA and serum ACE. An echocardiogram demonstrated an element of diastolic dysfunction, however, the heart on the whole functioned normally. He was seen by the rheumatology team and diagnosed with a vasculitis of unknown cause. He was treated with high dose IV methylprenisolone for three days followed by prednisolone 60 mg daily. The inflammatory markers returned to normal with an improvement in the rash and constitutional symptoms. Nerve conduction studies showed evidence of distal axonal sensorimotor neuropathy. The skin biopsy demonstrated features of a dense mixed perivascular infiltrate with moderately dense superficial and deep perivascular infiltrate comprising numerous eosinophils accompanied by neutrophils, lymphocytes and histiocytes. Lung function studies showed FEV1 is 2.49 (96%) with an FVC of 3.26 (108%), ratio of FEV1/FVC 76%. A diagnosis of eosinophilic vasculitis was made and the patient received pulsed intravenous cyclophosphamide for six months followed by a tapering dose of prednisolone and azathioprine was commenced. The foot drop and pleural effusions fully resolved with residual sensory changes in both feet. He remained well for over 18 months but then recently represented with pain, swelling and erythema over the right thigh. Blood tests again showed raised inflammatory markers and an eosinophilia. He was promptly treated with high dose steroids and switched to mycophenolate mofetil and may be a candidate for novel biologic therapy in the near future.

**Discussion:** This is an interesting and unusual case of a multisystem vasculitis that was challenging to diagnose and treat both in the short and long-term. This complex case demonstrates the need to consider a unifying diagnosis after appropriate investigations and the benefit of specialty involvement at an early stage. The recent relapse also raises questions about long term management strategies and the role of novel biologics.

**Key Learning Points:** This case illustrates the need for consideration of a wide differential diagnosis in cases of multisystem involvement with unusual neurology. Thorough investigations and involvement of specialists at an early stage can result in prompt treatment and an improved prognosis. This case also high-lights the importance of regular follow-up to detect early relapses and complications.

**Disclosure: G.K. Takhar:** None. **A. Porter:** None. **S. Allard:** None.

